# Incorporation of artificial intelligence into nursing research: A scoping review

**DOI:** 10.1111/inr.13013

**Published:** 2024-07-05

**Authors:** Yasin M. Yasin, Areej Al‐Hamad, Kateryna Metersky, Vahe Kehyayan

**Affiliations:** ^1^ Department of Nursing and Midwifery, Collage of Health Sciences University of Doha for Science and Technology Doha Qatar; ^2^ Daphne Cockwell School of Nursing, Daphne Cockwell School of Nursing Toronto Metropolitan University Toronto Canada; ^3^ Healthcare Management, College of Business University of Doha for Science and Technology Doha Qatar

**Keywords:** AI, artificial intelligence, benefit, challenge, nursing, research, review

## Abstract

**Background:**

The integration of artificial intelligence (AI) across different sectors, notably healthcare, is on the rise. However, a thorough exploration of AI's incorporation into nursing research, as well as its advantages and obstacles, is still lacking.

**Objective:**

The aim of this scoping review was to map the roles, benefits, challenges, and potentials for the future development and use of AI in the context of nursing research.

**Methods:**

An exhaustive search was conducted across seven databases: MEDLINE, PsycINFO, SCOPUS, Web of Science, CINAHL, Google Scholar, and ProQuest. Articles were additionally identified through manual examination of reference lists of the articles that were included in the study. The search criteria were restricted to articles published in English between 2010 and 2023. The Joanna Briggs Institute (JBI) approach for scoping reviews and the PRISMA‐ScR guidelines guided the processes of source selection, data extraction, and data presentation.

**Results:**

Twenty articles met the inclusion criteria, covering topics from ethical considerations to methodological issues and AI's capabilities in data analysis and predictive modeling.

**Conclusion:**

The review identified both the potentials and complexities of integrating AI into nursing research. Ethical and legal considerations warrant a coordinated approach from multiple stakeholders.

**Implication:**

The findings emphasized AI's potential to revolutionize nursing research, underscoring the need for ethical guidelines, equitable access, and AI literacy training to ensure its responsible and inclusive use.

## INTRODUCTION

Over the past decade, the integration of artificial intelligence (AI) into healthcare has continued to expand, offering unprecedented opportunities for the enhancement of patient care, healthcare research, and health systems management (Yu et al., 2018). Despite AI's demonstrated potential in improving diagnosis, treatment planning, and prediction of health outcomes through the analysis of large data (May, 2021), its application within the nursing profession is still in its formative stage (Ronquillo et al., 2021). Thus, the continued advancement of AI in the nursing profession promises to elevate the quality of care and operational efficiency (Ng et al., 2022; von Gerich et al., 2022).

AI technologies, including natural language processing, deep learning, machine learning, and predictive analytics, can be utilized to improve the efficiency and accuracy of nursing interventions, educate nursing students, and assist in nursing research (Robert, 2019). AI can be used to predict patient deterioration, streamline workflow, and reduce workload, thereby increasing the time for direct patient care (von Gerich et al., 2022). Additionally, AI algorithms can process and analyze data at speeds exceeding human capability. For instance, Poplin et al. (2018) utilized AI with deep learning capabilities to analyze the data of almost 300 000 patients quickly and accurately, interpreting genetic variations from high‐throughput sequencing data and processing thousands of genomes in a fraction of the time it would take by other software. Furthermore, the capability of AI for real‐time analysis holds exceptional value in patient monitoring and care. For instance, AI‐enhanced analysis of real‐time medical data could have played a pivotal role in the early detection, monitoring, and prevention of COVID‐19, offering a dynamic approach to managing public health crises (Hughes, 2020).

On the other hand, there are a number of significant barriers to the integration of AI into nursing research. These include a limited understanding of AI in healthcare and even society in general (Hulsen, 2023), lack of standardization in AI usage, ethical considerations, and fear of technology replacing human roles (Robert, 2019). Importantly, a recent systematic review of the application of AI in midwifery and nursing fields, which covered education, clinical practice, and policy, also acknowledged the scarcity of studies in these areas (O'Connor et al., 2023). The review, which concluded its search in July 2021, found no significant contributions specifically in nursing research, underscoring a persistent knowledge gap. It is therefore crucial to gain a clearer understanding of the current state of AI integration into nursing research and identify the potential challenges and opportunities AI presents.

It is pertinent to highlight how preceding reviews have paved the way for examining the impact of AI on nursing. For instance, the scoping review by Ng et al. (2022) provided a comprehensive overview of AI applications in enhancing clinical nursing practice, spotlighting its potential to elevate care quality by enhancing nurses’ readiness and effectiveness in managing patient conditions such as fall predication and wound management. The review underscored AI's role in clinical settings but fell short of exploring its implications for nursing research directly. Similarly, the von Gerich et al. (2022) scoping review delved into AI's clinical applications within nursing, focusing on its development, utilization, and performance. The review highlighted the technology's transformative potential in areas such as automated documentation and discharge planning, but once again omitted the impact AI can have on nursing research, particularly regarding how AI can support the advancement of nursing knowledge and evidence‐based practices. The scoping review by Buchanan et al. (2021) shifted the focus toward AI's influence on nursing education, both in academic and clinical settings, offering insights into how AI can shape learning outcomes and prepare nursing professionals for a technologically advanced healthcare environment. Although the review provided valuable perspectives on AI's educational benefits, it further delineated the need for a focused exploration of AI's role in nursing research itself. An initial search for previous reviews focused on the integration of AI into nursing research, conducted through JBI Evidence Synthesis and Open Science Framework, yielded no reviews in progress or ones that specifically addressed the incorporation of AI in nursing research.

There is a knowledge gap in comprehensively understanding how AI has been incorporated into nursing research and its potential development and use. Although bibliometric analysis provides valuable insights, focusing exclusively on quantitative metrics such as publication patterns and citation impact (Zupic & Čater, 2014), it falls short in offering a deeper understanding of the application of AI in nursing research. In contrast, a scoping review can comprehensively map a broad, emerging field and inform future research, policy, and educational, and nursing practice with its inclusive and flexible approach.

This scoping review aimed to fill this gap in the literature by exploring the range, extent, and nature of using AI in nursing research. The aim of this scoping review was to map the role, benefits, challenges, and potential for future development of AI in the context of nursing research. This scoping review sought to answer the following questions: How has AI been incorporated into nursing research? What are the associated challenges and benefits of AI incorporation into nursing research? Some examples of such incorporation can include AI techniques being used at every step of the research process from assisting with reviewing literature, to formulating hypotheses/questions, sampling and recruitment, ethics submission, collecting and analyzing data, and writing up and dissemination of results.

The outcomes of this scoping review will illuminate the current landscape of AI application within nursing research, thereby directly informing nursing researchers, educators, policy developers, leaders, and practitioners about the extent of AI integration, its practical benefits, and the challenges encountered with its use. This focused examination will serve as a foundational analysis for identifying specific areas where future research is needed, offering targeted recommendations for addressing the challenges of AI incorporation. Additionally, it aims to foster a dialogue on ethical considerations and effective strategies for integrating AI into nursing research, thus contributing to the development of informed guidelines that support the advancement of the nursing profession. Importantly, this review will bridge the gap between AI in nursing research and its consequential effects on patient care and nursing practice. By demonstrating how AI‐driven research can lead to innovative care models, improved patient outcomes, and more efficient healthcare services, we underscore the indirect yet significant impact that enhanced research methodologies can have in the clinical realm.

## METHODS

The Joanna Briggs Institute (JBI) approach for scoping review and the PRISMA‐ScR guidelines guided the processes of source selection, data extraction, and data presentation (Peters et al., 2020). The review protocol was registered in the Open Science Framework (Yasin et al., 2024).

### Inclusion criteria

#### Population

In the context of a scoping review, the population refers to the target group under consideration (Peters et al., 2020). For this scoping review, the population included nursing researchers, educators, practitioners, and policymakers who were involved in incorporating AI into nursing research. Papers centering on other healthcare professionals or the general public, and those researchers who were not involved in AI integration into nursing research, were excluded.

#### Concept

The concept in a scoping review refers to the main idea or phenomena of interest (Peters et al., 2020). The concept for this review is AI. It can be defined as the methods employed to enable computers to learn, think, perceive, deduce, communicate, and make choices at a level that is comparable with or surpasses human capabilities (Robert, 2019). AI encompasses capabilities such as natural language processing, deep learning, machine learning, and automated reasoning (Russell & Norvig, 2010). Papers that reported the application of AI technology to nursing research were included. Papers that did not focus on AI or its specific applications were excluded.

#### Context

The context is the setting or circumstance in which the concept occurs (Peters et al., 2020). For this review, the context included all settings where AI was incorporated into nursing research, irrespective of the country, type of institution, or levels of care. Papers that did not specifically discuss the utilization of AI techniques within the context of nursing research were excluded. Papers that focused on management, education, or practice application were also excluded.

#### Types of resources

The type of resources in this scoping review included primary research and review articles, editorials, theses, dissertations, conference proceedings, reports, and other gray literature discussing the incorporation of AI into nursing research. Publications after 2010 were included because AI has rapidly evolved in the past 13 years (Dormehl, 2019). Only English‐language papers were included as members of the research team had limited proficiency in other languages. Websites and blogs were excluded to maintain higher academic and research standards. See Table 1 for eligibility criteria.

**TABLE 1:** Inclusion and exclusion criteria.

	Inclusion criteria	Exclusion criteria
Population	Nursing researchers, educators, practitioners, and policymakers	Other healthcare professionals or the general public
Concept	Incorporation of AI into nursing research	Studies not centered on the use of AI in nursing studies
Context	All settings where AI is incorporated into nursing research	Studies that do not discuss the application of AI within the context of nursing research such as education or practice
Type of resources	Primary research articles, review articles, editorials, theses, dissertations, reports, and other gray literature written in the English language and published after 2010	Websites, blogs

### Search strategy

The search strategy for this scoping review was carried out through three extensive phases. The first phase, known as “initial identification,” commenced with a preliminary exploration in CINAHL (see Appendix A for search strategy). During this initial phase, the primary focus was on scrutinizing the keywords incorporated within the title and abstract, along with the indexed terms utilized to describe these articles. This methodological step assisted in constructing a well‐rounded search strategy to guide the next steps of the review.

Moving into the second phase, termed the “comprehensive search,” the review process was broadened to apply the previously identified index terms and keywords uniformly across all relevant databases and sources. This ensured an exhaustive and extensive collection of data on the topic, encompassing as many sources as possible to gather a complete understanding of the issue at hand. In the final phase, called the “reference list review,” a thorough examination of the reference lists linked with the selected studies identified during the screening process was undertaken. This stage uncovered more relevant studies that were not discovered during the first two phases.

The review delved into the following databases: MEDLINE and PsycINFO via OVID, SCOPUS via Elsevier, Web of Science via Clarivate, and CINAHL via EBSCOhost. An inclusive strategy that wrapped all the identified keywords and indexed terms was customized for each database. Additionally, the reference lists of all sources of evidence included in this review were meticulously screened to uncover any potential additional studies. To ensure an exhaustive search, gray literature sources including ProQuest (Dissertation and Theses Global), and broader platforms including Google Scholar were included in the search strategy. This inclusion aimed at capturing every possible piece of information pertaining to the topic of interest to provide a holistic review.

### Study selection

Upon completion of the search phase, all discovered records were assembled and imported into the Endnote reference management software version 20 (The EndNote Team, 2013). This software automatically identified and eliminated any duplicate and retracted entries. Subsequently, these records were transferred into JBI SUMARI, a web‐based tool designed for reviewing articles (Munn et al., 2018). This platform was utilized by two independent reviewers (AA and KM) who screened each title and abstract against the preset inclusion criteria for the review.

**FIGURE 1:**
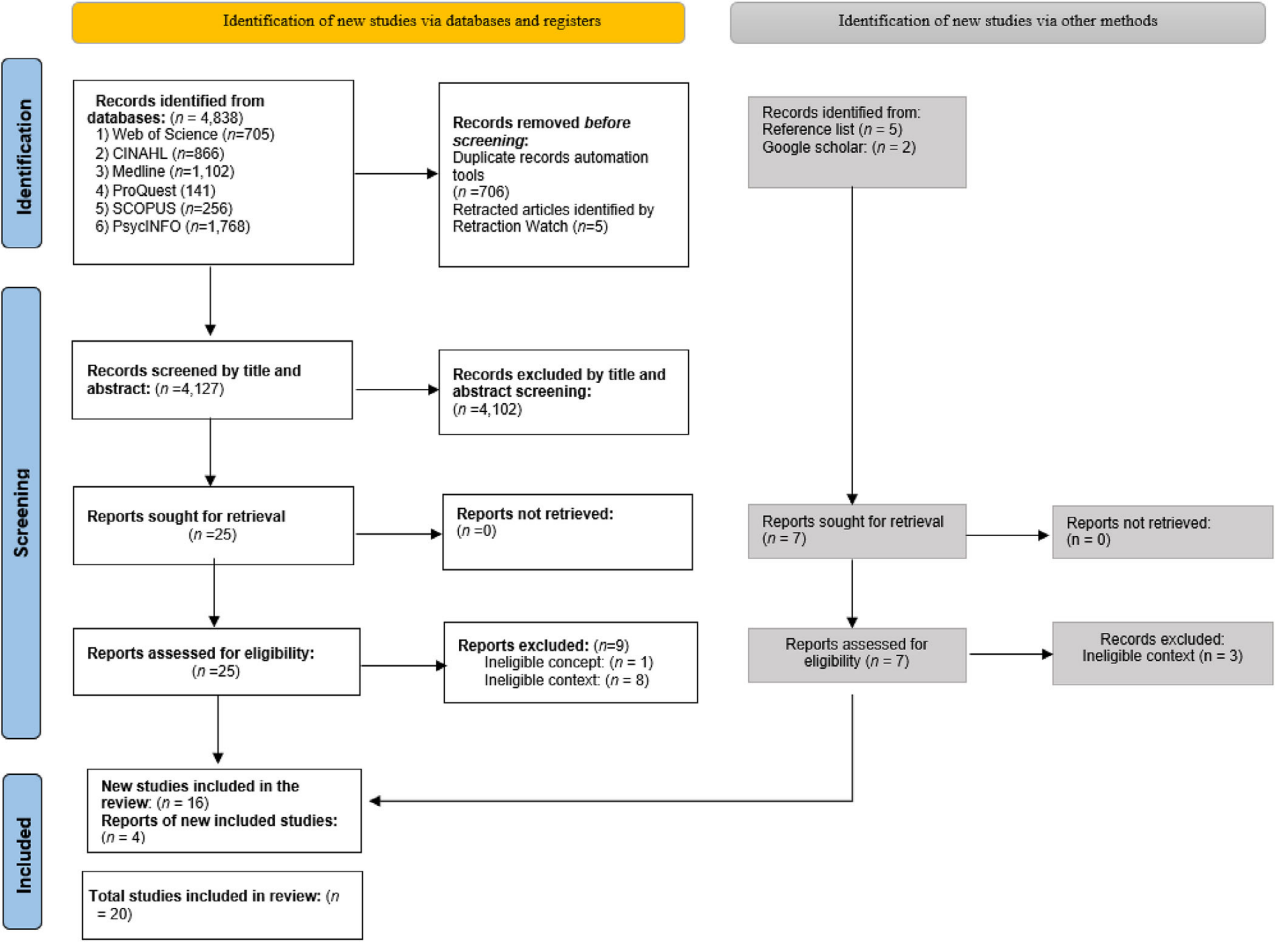
PRISMA flow diagram.

Then, the full texts of potential sources deemed relevant in the preliminary screening were retrieved for further evaluation in JBI SUMARI by two independent reviewers (AA and KM). At this stage, if certain sources failed to meet the inclusion criteria, they were excluded with specific reasons for exclusion diligently recorded and reported in the final scoping review. When disagreements between reviewers arose at any stage of the selection process, they were resolved via detailed discussion or consultation with a third reviewer (YY). This approach ensured the impartiality and objectivity of the study selection process. The outcomes of the search process and subsequent study selection were fully reported in the completed scoping review. Additionally, the results were visualized in a PRISMA diagram to provide a clear representation of the review progress (see Figure 1).

### Data extraction

For the sake of consistency, two independent reviewers (VK and YY) carried out the data extraction from each article chosen for the scoping review. The JBI SUMARI's standardized data extraction tool was utilized for this process with slight modifications to meet the purpose of this review. The information extracted encompassed specific details regarding the AI concept, nursing research study methods, context, and critical findings that aligned with the goals of the review. This extraction aimed to outline the context, methodology, and description of the main results related to nursing research utilizing AI. Discrepancies between the reviewers during this stage were resolved through discussion or by consulting a third reviewer (AA).

### Data analysis and presentation

The approach for data analysis relied on inductive content analysis (Elo & Kyngäs, 2008). Two independent reviewers (VK and YY) engaged in tallying report characteristics and the count of articles that discussed how AI was incorporated into nursing research, along with the identified challenges and benefits of its use. These data were subsequently subjected to basic coding conducted by the two independent reviewers (VK and YY). The coding process involved a detailed examination of data (such as academic articles, reports, and other relevant documents identified in a scoping review) to identify significant codes related to the population, concept, and context of our paper. The codes were then grouped into themes based on their similarities or differences. The process was iterative and dynamic, with codes being continuously refined and adjusted as more data were analyzed.

To ensure the consistency and accuracy of the coded data, a process of comparison was implemented among team members. Any disagreements were resolved through either consensus among the team or consultation with the third reviewer (AA). Then, the coded data were organized into distinct categories. The number of articles that fell under each category was compiled and presented in the extraction table shown in Appendix B.

## RESULTS

The initial literature search yielded 4838 records for consideration. Of these, we removed 706 duplicate entries and five articles flagged by Retraction Watch (through the Endnote reference management software), leaving 4127 records for initial review. Retraction Watch is an online platform that monitors the withdrawal of articles in scientific and academic fields. During this preliminary phase, the titles and abstracts were scrutinized for their relevance to the study's scope. This screening resulted in the selection of 25 records for in‐depth, full‐text evaluation. Out of these, nine were subsequently excluded for reasons such as contextual ineligibility (eight articles) and concept mismatch (one article). An additional four articles were incorporated into the review following a targeted search on Google Scholar and a manual review of the reference lists from the included articles. Ultimately, 20 studies met the criteria for inclusion in the final scoping review. See Figure 1 the PRISMA diagram.

### Included studies

The included articles in this scoping review spanned a publication date range from January 2010 to August 2023, highlighting the dynamic development of AI within nursing research. Ethical and legal considerations surrounding AI were explored in eight commentaries and editorials (Abdulai & Hung, 2023; Dave et al., 2023; Dwivedi et al., 2023; Lyon, 2023; Miao & Ahn, 2023; Moons & Van Bulck, 2023; Ruppar, 2023; Yasin & Al‐Hamad, 2023). Two bibliometric reviews offered a statistical lens into AI's emerging research hotspots (Duan & Zhao, 2023; Shi et al., 2023), whereas two systematic reviews synthesized the existing literature to offer structured insights into AI‐associated activities in nursing (Hwang et al., 2022; Kikuchi, 2020). Five quantitative studies emphasized the predictive capabilities of AI in different healthcare settings (Bose et al., 2019; Ladstatter et al., 2010; Ladstatter et al., 2016; Linz et al., 2021; Moen et al., 2022). On the other hand, two mixed‐method studies (Brehon et al., 2023; Cai et al., 2023) and a single qualitative study (Byon et al., 2023) highlighted AI's role in data analysis, especially in unstructured qualitative data.

The included papers encompassed a wide variety of AI concepts. ChatGPT was reported in eight papers (Abdulai & Hung, 2023; Dave et al., 2023; Dwivedi et al., 2023; Lyon, 2023; Miao & Ahn, 2023; Moons & Van Bulck, 2023; Ruppar, 2023; Yasin & Al‐Hamad, 2023); machine learning was covered in four papers (Bose et al., 2019; Brehon et al., 2023; Cai et al., 2023; Moen et al., 2022); natural language processing was a focal point in three papers (Byon et al., 2023; Cai et al., 2023; Moen et al., 2022); and finally, artificial neural networks were the subject of investigation in two quantitative articles (Ladstatter et al., 2010; Ladstatter et al., 2016).

### Benefits of AI in nursing research

**FIGURE 2:**
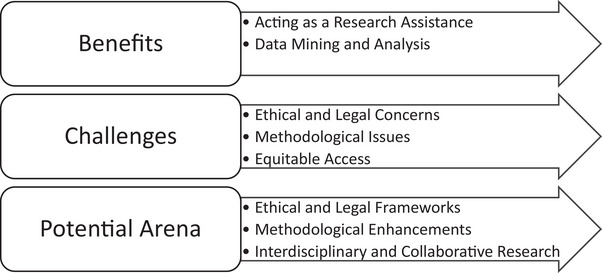
Summary of the findings for incorporation AI in nursing research.

The findings of this scoping review were categorized into benefits, challenges, and potential arenas for AI use in nursing research. See Figure 2 for the summary of each finding category. To begin, the benefits of AI in nursing research were extensively explored. Notably, five articles highlighted the role of AI tools, specifically ChatGPT, in serving as a research assistant. These tools assisted in various research assistant activities such as literature reviews and manuscript drafting (Dave et al., 2023; Dwivedi et al., 2023; Moen et al., 2022; Moons & Van Bulck, 2023; Yasin & Al‐Hamad, 2023). Additionally, the role of AI in data mining was emphasized in five articles, focusing particularly on its capability in automating the screening of article abstracts (Hwang et al., 2022; Moen et al., 2022), facilitating clinical trial recruitment (Dave et al., 2023; Linz et al., 2021), and managing data (Shi et al., 2023). The potential of AI to expedite the research process and enhance the quality of scholarly publications was also underscored and was especially beneficial for authors for whom English was not their first language (Lyon, 2023; Yasin & Al‐Hamad, 2023).

Several articles pointed out the usage of AI in data analysis (Hwang et al., 2022; Lyon, 2023; Yasin & Al‐Hamad, 2023). Four articles indicated AI's superior predictive abilities, particularly in capturing nonlinear relationships, which can be considered an advancement for theory development (Bose et al., 2019; Kikuchi, 2020; Ladstatter et al., 2010; Ladstatter et al., 2016). Two other articles specifically reported the use of AI in risk prediction and data analysis (Duan & Zhao, 2023; Shi et al., 2023). Five studies highlighted the utility of natural language processing and machine learning in analyzing clinical notes and qualitative data, offering methods for deriving information from large sets of unstructured data (Bose et al., 2019; Brehon et al., 2023; Byon et al., 2023; Cai et al., 2023; Dwivedi et al., 2023). Lastly, one article focused on the flexibility of AI tools in opening new avenues for innovative interdisciplinary research, enabling researchers to explore new questions and methodologies (Miao & Ahn, 2023).

### Challenges of AI in nursing research

The challenges associated with the integration of AI in nursing research were thoroughly examined in the reviewed papers. Ethical and legal considerations were a major theme highlighted in five papers (Dave et al., 2023; Dwivedi et al., 2023; Lyon, 2023; Ruppar, 2023; Yasin & Al‐Hamad, 2023). Two papers specifically raised concerns about academic dishonesty and copyright infringement (Abdulai & Hung, 2023; Dave et al., 2023). Additionally, five papers raised questions about the quality and accuracy of AI‐generated outputs (Dave et al., 2023; Dwivedi et al., 2023; Kikuchi, 2020; Moons & Van Bulck, 2023; Ruppar, 2023). One article pointed out the possibility of errors or biases in writing, attributing them to less critical engagement with literature and data (Yasin & Al‐Hamad, 2023).

Methodological issues were cited in four papers that addressed limitations such as small sample sizes (Brehon et al., 2023; Kikuchi, 2020), data imbalance (skewed data set), and problems with sensitivity and specificity (Cai et al., 2023; Moen et al., 2022). One article specifically identified the absence of structured data as a limitation in AI analyses of clinical notes (Brehon et al., 2023). Another article criticized artificial neural networks for their inability to explain the impact of specific predictor variables on outcomes (Ladstatter et al., 2016).

Equitable access to AI tools also emerged as a significant issue, particularly regarding the potential for creating disparities in research opportunities among researchers with varying levels of AI literacy (Miao & Ahn, 2023). Two papers cautioned that the increasing role of AI might make it challenging for junior researchers to establish independent research programs, given the technical and financial barriers to accessing cutting‐edge AI tools (Dwivedi et al., 2023; Miao & Ahn, 2023).

### Potential arena for AI in nursing research

The suggestions for enhancing the role of AI in nursing research were articulated across various dimensions in the included papers. Ethical and legal considerations were particularly emphasized, with four papers advocating for the development of clear guidelines for the ethical and responsible use of AI tools such as ChatGPT (Abdulai & Hung, 2023; Dwivedi et al., 2023; Moons & Van Bulck, 2023; Yasin & Al‐Hamad, 2023). One paper called for greater transparency from authors in their use of AI tools (Dave et al., 2023), and another suggested that both authors and journal editors need specific guidance on how to ethically and scientifically incorporate AI tools into research and publication processes (Lyon, 2023).

On the methodological front, three papers stressed the importance of designing studies with larger and more diverse sample sizes to address limitations identified in prior research (Brehon et al., 2023; Cai et al., 2023; Moen et al., 2022). Calls for further validation of AI research results were made in another three articles, particularly when the findings were intended for clinical applications (Ladstatter et al., 2010; Moons & Van Bulck, 2023; Ruppar, 2023). One paper placed additional emphasis on the importance of structured approaches to data collection (Brehon et al., 2023), whereas another articulated the need for utilizing more advanced machine learning techniques (Cai et al., 2023). Three further papers highlighted the necessity for additional research to advance the field of AI use in research (Bose et al., 2019; Byon et al., 2023; Dave et al., 2023). One paper underscored the value of interdisciplinary approaches and the development of data sets suitable for machine‐based analysis as key steps forward (Kikuchi, 2020). Lastly, two papers advocated for fostering a collaborative and inclusive research environment where AI complements rather than replaces human intelligence (Dwivedi et al., 2023; Yasin & Al‐Hamad, 2023).

## DISCUSSION

This scoping review commenced with an initial pool of 4838 papers, eventually narrowing down to 20 papers that met the inclusion criteria. These papers, published between January 2010 and August 2023, offered diverse perspectives on the roles, benefits, challenges, and potential enhancements of using AI in nursing research. The included papers covered a range of topics from ethical considerations to methodological issues and the capabilities of AI in data analysis and predictive modeling. After a thorough search of the literature, this is the first review to focus specifically on the use of AI in nursing research.

### Benefits of AI in nursing research

The benefits of using AI in nursing research align closely with broader technological trends in healthcare and other academic disciplines. For instance, the use of AI tools for tasks such as literature reviews and manuscript drafting has been echoed in other fields, highlighting the universal value of AI in enhancing research efficiency (Wang et al., 2023). This is especially significant for nonnative English speakers, as AI can help mitigate language barriers, making scholarly work more accessible (Zenni & Andrew, 2023).

The data analysis capabilities of AI signify an advancement not just for nursing but also for healthcare research as a whole (Shaheen, 2021). These capabilities are part of a growing trend where natural language processing and machine learning are leveraged to analyze large and complex data sets, offering innovative approaches for deriving valuable insights (Le Glaz et al., 2021). Nonetheless, the review findings suggest that some skepticism remains regarding the reliability and validity of AI‐generated data analysis. Therefore, there is an urgent requirement for additional studies focused on improving the validity and reliability of AI methods in data analysis.

The flexibility of AI tools, particularly in fostering interdisciplinary research, is in harmony with broader academic discourse that advocates for the integration of various fields of study to provide a more holistic approach to research (Kusters et al., 2020). This push for interdisciplinary methods and the cultivation of a collaborative research atmosphere echoes prevailing trends in academic dialogue.

### Ethical and regulatory considerations in AI integration

The diversity of the articles included in this study not only underscores the complexity of integrating AI into nursing research but also signifies its potential breadth of application. This diversity is a double‐edged sword; while it allows for a multitude of research opportunities, it also complicates the ethical and regulatory landscapes. The preponderance of articles focusing on ethical and legal considerations is indicative of the broader ongoing debates in healthcare ethics and technology governance (Abdulai & Hung, 2023; Dwivedi et al., 2023). This focus is not unique to nursing research but is a critical part of the larger discourse surrounding digital health innovations and their integration into healthcare systems (Dhirani et al., 2023). Addressing ethical issues will require a concerted effort from researchers, ethicists, and policymakers alike. The notion that AI should augment rather than replace human expertise is a key point discussed in AI ethics literature (Crawford & Calo, 2016). The integration of AI in research and clinical settings necessitates a balanced approach that maximizes benefits while minimizing risks, a viewpoint supported by scholars advocating for responsible AI use (Floridi et al., 2018).

### Implications

The findings from this scoping review have several important implications for research, policy, and education in the field of nursing and healthcare at large. For research, tools such as ChatGPT and Scispace have been shown to serve as valuable assistants in conducting literature reviews, drafting manuscripts, and managing data. This suggests an evolving research model where AI complements human efforts, potentially freeing researchers to focus on more complex analytical tasks. Moreover, the capabilities of AI in data mining, clinical trial recruitment, and analysis hold the promise of streamlining research processes, thereby offering pathways to insights that are both timelier and more efficient in terms of resource use. Furthermore, there is a need for further studies to validate and improve the reliability of AI methodologies in data analysis. Equitable access to AI technologies is emerging as a critical issue, with disparities in access potentially exacerbating existing inequities within the research community. This calls for targeted efforts to democratize AI technology, ensuring researchers at all levels and from diverse backgrounds can benefit from AI tools. Such efforts might include training programs to build AI literacy, funding opportunities to acquire necessary technology, and partnerships between academic institutions and technology companies to provide access to cutting‐edge AI resources.

From a policy perspective, the prevalence of ethical and legal considerations in the existing literature points to the need for regulatory frameworks that can address the complexities introduced by AI. Policymakers should collaborate with ethicists, clinicians, and researchers to develop guidelines that ensure the responsible deployment of AI tools in nursing research. It is crucial for the academic community to develop standardized protocols and guidelines for the ethical use of AI, particularly as it becomes an increasingly indispensable tool in research settings. This aligns well with broader debates on healthcare ethics and technology governance that necessitate a multistakeholder approach.

In terms of education, this review accentuates the value of AI in streamlining research processes and enabling intricate data analyses. However, to harness these advantages to their full extent, there exists a pressing need for comprehensive training programs aimed at equipping nursing researchers with the requisite skills for effective AI utilization. The imperative for AI literacy transcends mere tool development and adoption, extending into equitable and effective application across various levels of healthcare research and practice.

## LIMITATIONS

The primary limitations of this review include its focus solely on English‐language articles, potentially excluding pertinent studies in other languages. Additionally, the inclusion of diverse paper types complicated the process of forming generalized conclusions. Furthermore, the authors recognize that additional research on this topic may have emerged following the initial search conducted in September 2023. Despite the relevance of such studies, practical constraints including time and limited resources prevented the undertaking of an updated search. This acknowledgment reflects our commitment to transparency regarding the scope and timing of our review, while also underscoring the dynamic and rapidly evolving nature of research on the topic of AI within nursing research. Despite these limitations, the review provided valuable insights into the current landscape of AI in nursing research, highlighting areas for future investigation and development.

## CONCLUSION

This scoping review provided a comprehensive overview of the burgeoning field of AI use in nursing research, examining its benefits, challenges, and potential areas for future development. The review identified key areas where AI tools have shown promise in enhancing research efficiency, facilitating complex data analysis, and fostering interdisciplinary collaboration. However, it also highlighted pressing ethical and legal considerations that warrant a coordinated approach from researchers, ethicists, and policymakers. With continued advancements in AI technologies, the need for a balanced and ethical integration of these tools into nursing research becomes increasingly imperative.

## AUTHOR CONTRIBUTIONS

*Study design*: Yasin M. Yasin and Areej Al‐Hamad. *Data collection*: Areej Al‐Hamad and Kateryna Metersky. *Data analysis*: Yasin M. Yasin and Vahe Kehyayan. *Study supervision*: Yasin M. Yasin. *Manuscript writing*: Yasin M. Yasin, Areej Al‐Hamad, Kateryna Metersky, and Vahe Kehyayan. *Critical revisions for important intellectual content*: Yasin M. Yasin, Areej Al‐Hamad, Kateryna Metersky, and Vahe Kehyayan.

## CONFLICT OF INTEREST STATEMENT

The authors declare no conflict of interest.

## FUNDING INFORMATION

This research received no specific grant from any funding agency in the public, commercial, or not‐for‐profit sectors.

## ETHICS STATEMENT

As this is a review study and there were no human participants, we did not seek ethical approval.

## Supporting information

Supporting Information

Supporting Information

## Data Availability

The data that were used to support the findings of this scoping review can be found within the text of the cited references included in this study.
