# Supplementary material for: Incorporation of artificial intelligence into nursing research: A scoping review
Source: Int Nurs Rev. 2024 Jul 5;72(1):e13013. doi: 10.1111/inr.13013 (PMC11741909; doi:10.1111/inr.13013)
Supplement: Supplementary file 1 — Supporting Information [file INR-72-0-s002.docx]

**Appendix A: CINAHL search strategy**

| **Component** | **#** | **Query** | **Retrieved record** |
| --- | --- | --- | --- |
| **Population** | S1 | (MM "Research Nurses+") OR (MM "Clinical Research Nurses") | 2,009 |
|  | S2 | nurs* OR "nurse researcher" OR "nurse educator" OR "nurse practitioners" OR "nurse administrator" | 1,008,994 |
|  | S3 | S1 OR S2 | 1,008,994 |
| **Concept** | S4 | (MH "Artificial Intelligence+") OR "Artificial Intelligence" | 33,060 |
|  | S5 | “Artificial intelligence” OR “ai” OR “a.i” or “machine learning” OR “deep learning” OR “neural network” OR “natural language processing” | 44,877 |
|  | S6 | S4 OR S5 | 59,217 |
| **Context** | S7 | (MM "Research+") OR (MM "Research, Nursing") OR (MM "Research Support+") | 338,462 |
|  | S8 | research OR study OR method OR qualitative OR quantitative | 4,117,442 |
|  | S9 | S7 OR S8 | 4,160,948 |
|  | S10 | S3 AND S6 AND S9 | 1,893 |
