# Supplementary material for: Incorporation of artificial intelligence into nursing research: A scoping review
Source: Int Nurs Rev. 2024 Jul 5;72(1):e13013. doi: 10.1111/inr.13013 (PMC11741909; doi:10.1111/inr.13013)
Supplement: Supplementary file 2 — Supporting Information [file INR-72-0-s001.docx]

**Appendix B: Extraction table**

| **Study** | **Country & sample** | **Context** | **Methodology** | **AI concept** | **Description of main results related to nursing research** |
| --- | --- | --- | --- | --- | --- |
| Abdulai A, Hung L. 2023. | NA | Ethical/Legal discussion | Commentary/ editorial | AI tools, ChatGPT | - **Benefits:** Not reported - **Challenges:**  1. Facilitate academic dishonesty.  - **Suggestions:**  1. Developing clear guidelines and promoting academic integrity 2. AI tools like ChatGPT should be used responsibly and ethically to enhance the quality of research |
| Bose E. Maganti S. Bowles KH. Brueshoff BL. Monsen KA. 2019 | USA  (*n*=756) | Research study | Quantitative research | Machine learning | - **Benefits:**  1. AI can improve prediction performance and can inform more efficient documentation 2. Enable selection of important features based on scoring metrics. 3. Machine learning methods have far-reaching applications in any application that requires the reduction of features in big data.  - **Challenges:** Not reported - **Suggestions:**  1. Further research is needed to determine whether the research findings apply in other contexts |
| Brehon K, Carriere J, Churchill K, Loyola-Sanchez A, Papathanassoglou E, MacIsaac R, et al. 2023. | Canada  (*n*= 412) | Research study | Mixed method study | Artificial Intelligence/Machine Learning (AI/ML) using the Apache cTakes NLP system | - **Benefits:**  1. AI/ML can be used to analyze clinical notes in telephone calls.  - **Challenges:**  1. AI/ML analyses were limited by the fact that the clinical notes were unstructured. 2. The AI/ML analyses were limited by sample size.  - **Suggestions:**  1. More structured notes with identifiable features and a larger dataset would allow for more rigorous AI/ML analyses to be conducted. |
| Byon HD, Harris C, Crandall M, Song J, Topaz M. 2023. | USA  (*n*=600,00) | Research study | Qualitative study | Machine-learning-based natural language processing algorithms | - **Benefits:**  1. Data analysis: Natural language processing can be an effective tool to augment formal reporting by capturing violence incidents from large volumes of clinical notes.  - **Challenges:** Not reported - **Suggestions:**  1. The study recommended developing natural language processing and increasing the amount of data available for research |
| Cai YR, Leveille SG, Andreeva O, Shi L, Chen P, You TJ. 2023. | USA  (*n*=765) | Research study | Mixed method study | Machine learning: Natural language processing (N-grams) | - **Benefits:**  1. Data analysis of narrative responses from open-ended questions. 2. Obtain extensive information from qualitative data.  - **Challenges:**  1. Other machine learning approaches may better depict fall circumstances based on narrative fall data.  - **Suggestions:**  1. Using digital video cameras or real-time monitors and using machine learning approaches such as artificial neural networks to process the data may provide important information. 2. Need for larger sample sizes and longer follow-up intervals for managing and coding the extensive text data. |
| Dave T, Athaluri SA, Singh S. 2023. | NA | Ethical/Legal discussion | Commentary/ editorial | AI tools, ChatGPT | - **Benefits:**  1. Writing scientific literature with eloquent vocabulary 2. Can be used as a rapid search engine. 3. Assist in topic selection. 4. Save time and effort by searching and analyzing literature. 5. Aid in clinical trial recruitment by analyzing large amounts of patient data to identify individuals who meet the trial’s eligibility criteria.  - **Challenges:**  1. Infringement of copyright laws, medico-legal complications, and the potential for inaccuracies or prejudices in the generated content 2. Sometimes inaccurate, biased or harmful results  - **Suggestions:**  1. Advanced systems should be developed which can focus on identifying even the small manipulation in the data done by the ChatGPT 2. Authors should include how they used ChatGPT as a tool to assist them in their research. |
| Duan SY, Zhao Y. 2023. | NA | Bibliometric review | Bibliometric analysis and science mapping | Machine learning, Deep learning, Deep learning, Natural language processing, Artificial Intelligence | - **Benefits:**  1. ‘‘electronic health record”, ‘‘risk prediction” and ‘‘supervised machine learning” can be regarded as the latest emerging AI research hotspots.  - **Challenges:** Not reported - **Suggestions:** Not reported |
| Dwivedi YK, Kshetri N, Hughes L, Slade EL, Jeyaraj A, Kar AK, et al. 2023. | NA | Ethical/Legal discussion | Commentary/ editorial | AI tools, ChatGPT | - **Benefits:**  1. Improve writing: give useful introductions to topics, guiding the human researcher as to which ones to follow up. 2. Work as a research assistant 3. Reviewing and analyzing huge amount of data 4. Journal/conference editors and reviewers may also be able to use generative AI to screen submitted manuscripts.  - **Challenges:**  1. Ethical issues 2. Legal issues 3. Transparency and explainability. 4. Dependence on technology 5. Loss of jobs. 6. Lack of originality.  - **Suggestions:**  1. Multiple stakeholders can, and must, make positive contributions towards designing and deploying more responsible and ethical AI. 2. Researchers should explore how best humans and AI can work together to maximize the opportunities and benefit of generative AI, as well as minimizing negative impact and risks. |
| Hwang GJ, Chang PY, Tseng WY, Chou CA, Wu CH, Tu YF. 2022. | NA | Systematic review | PRISMA guidelines | Artificial intelligence–associated nursing activities | - **Benefits:**  1. Data analysis 2. Data mining  - **Challenges:** Not reported - **Suggestions:** Not reported |
| Kikuchi R. 2020. | NA | Systematic review | No specific guideline reported | “artificial intelligence,” “machine learning,” “deep learning,” “neural networks,” | - **Benefits:**  1. Measuring outcomes 2. Better performance than traditional statistical analysis  - **Challenges:**  1. Limited sample size 2. Doubts about the results 3. Would not be able to cope with emergency cases 4. Enormous amounts of data are required to train AI technology.  - **Suggestions:**  1. To extend the application of AI technology to nursing science, an interdisciplinary approach is indispensable. 2. Construction of a dataset suitable for machine-based analysis is also key |
| Ladstätter F. Garrosa E. Moreno B, Ponsoda V., Aviles J. Dai J. 2016 | China  (*n*=465) | Research study | Quantitative research | Artificial neural networks (ANN) | - **Benefits:**  1. ANN has superior predictive ability compared to multiple regression method for data analysis.  - **Challenges:**  1. The lack of capacity of ANNs to analyze the exact impact of a particular predictor variable on the output variable.  - **Suggestions:** Not reported |
| Ladstätter F. Garrosa E. Badea C. Moreno B. 2010 | Spain  (*n*=462) | Research study | Quantitative research | Artificial neural networks (ANN) | - **Benefits:**  1. ANN has superior predictive ability to capture non-linear relationships, which is relevant for theory development.  - **Challenges:** Not reported - **Suggestions:**  1. Future investigations would be interesting to include a comparison of multiple regression, in which interactions between predictor variables are taken into account. |
| Linz N, Ter Huurne DB, Langel K, Ramakers IH, König A. 2021. | Netherlands  (*n*=140) | Conference presentation | Quantitative study | Machine learning models | - **Benefits:**  1. AI empowered recruitment for clinical trials  - **Challenges:** Not reported - **Suggestions:** Not reported |
| Lyon D. 2023. | NA | Ethical/Legal discussion | Commentary/ editorial | Large Language Model Chatbots | - **Benefits:**  1. ChatGPT and other chatbots may facilitate scholarly publication and increase equity in access to publication. (For authors who speak English as an additional language) 2. ChatGPT can be leveraged to improve the efficiency and accuracy of the writing process. 3. ChatGPT also has potential uses in data analysis.  - **Challenges:**  1. Multiple ethical and legal concerns  - **Suggestions:**  1. Authors need guidance about how to use these tools in a scientifically defensible manner. 2. Editors need to consider a middle ground that permits the use of chatbots in appropriate parts of manuscript |
| Miao H, Ahn H. 2023. | NA | Ethical/Legal discussion | Commentary/ editorial | AI tools, ChatGPT | - **Benefits:**  1. Implies potential for innovative interdisciplinary research in nursing.  - **Challenges:**  1. Researchers with better knowledge of and access to AI technologies may have an advantage in terms of research productivity and quality, potentially creating disparities within the field. 2. If AI becomes a dominant factor in research, assessing the contributions of human researchers becomes more complex. 3. The increasing role of AI may make it challenging for junior researchers to establish independent research programs, given the technical and financial barriers to accessing cutting-edge AI tools.  - **Suggestions:** Not reported |
| Moen H, Alhuwail D, Björnea,J, Block L, Celin S, Jeon E, et al. 2022. | NA  (*n*=4,186) | Research study | Quantitative study | Natural Language Processing | - **Benefits:**  1. AI can be used to automate the screening of article abstracts with a nursing relevance when a training set exists.  - **Challenges:**  1. Data Imbalance, sensitivity, and specificity issues  - **Suggestions:**  1. Needs for further studies to improve the results of AI |
| Moons P. Van Bulck L. 2023 | NA | Ethical/Legal discussion | Commentary/ editorial | AI tools: ChatGPT, | - **Benefits:**  1. Research Assistance: Keeping up to date with the latest research and guidelines by summarizing articles, papers, and other sources of information.  - **Challenges:**  1. It remains crucial that the output is evaluated by professionals for accuracy and completeness.  - **Suggestions:**  1. It’s important for nurses to use ChatGPT in accordance with professional standards and guidelines. 2. It is important to review any output generated by ChatGPT for accuracy and completeness before using research findings in patient care. |
| Ruppar T. 2023. | NA | Ethical/Legal discussion | Commentary/ editorial | AI tools, ChatGPT | - **Benefits:** Not reported - **Challenges:**  1. Quality and accuracy of output from AI engines are going to be highly variable based on the engine used and the input prompts. 2. Legal and ethical concerns  - **Suggestions:**  1. We should not rush to use new technology in generating the scientific publications that constitute the evidence base for healthcare practice |
| Shi J, Wei S, Gao Y, Mei F, Tian J, Zhao Y, et al. 2023. | NA | Bibliometric Review | Bibliometric analysis and science mapping | Machine learning,  Natural language processing, Artificial neural network  Deep learning, | - **Benefits:**  1. Application of AI technology in information data management 2. Construction of risk prediction models  - **Challenges:** Not reported - **Suggestions:** Not reported |
| Yasin YM, Al-Hamad A. 2023. | NA | Ethical/Legal discussion | Commentary/ editorial | AI tools such as ChatGPT, RapidMiner, Copilot by SCISPACE and Iris.ai | - **Benefits:**  1. Data analysis 2. Serve as research assistant. 3. Expedite the literature review. 4. Drafting manuscripts 5. Overcome language barriers.  - **Challenges:**  1. Less critical engagement with literature and data analysis 2. Introduce errors or biases in writing.  - **Suggestions:**  1. Mindful and responsible use of AI tools 2. AI tools should be used judiciously, supplementing human intelligence rather than replacing it. 3. Authors to ensure its correctness, persuasiveness, and ethical uprightness. |
